# Age Differences in Personality Structure

**DOI:** 10.1093/geroni/igab046.2158

**Published:** 2021-12-17

**Authors:** Joshua Jackson, David Condon, Emorie Beck

**Affiliations:** 1 Washington University in St. Louis, St. Louis, Missouri, United States; 2 University of Oregon, Eugene, Oregon, United States; 3 Northwestern University Feinberg School of Medicine, Chicago, Illinois, United States

## Abstract

Most investigations in the structure of personality traits do not adequately address age, as few studies look at the structure of personality traits a-theoretically, instead presupposing a theoretical structure e.g., Big Five. As a result, the relationship among indicators within a trait (coherence) are often highlighted but relationships across traits (differentiation) are not thoroughly examined. Using a large-scale sample of 369,151 individuals ranging in age from 14 to 90, the present study examines whether personality indicators show differential relationships as a function of age. Results indicate that coherence shows few changes across the lifespan, while differentiation weakens across adulthood into old age. These finding suggest that Big Five indicators only parallel the Big Five structure among young but not older adults. Thus, using standard Big Five personality trait assessments in older adults may, at best, not reflect reality and, at worse, undermine the predictive utility of personality traits.

